# Mapping genes for resistance to stripe rust in spring wheat landrace PI 480035

**DOI:** 10.1371/journal.pone.0177898

**Published:** 2017-05-19

**Authors:** Jinita Sthapit Kandel, Vandhana Krishnan, Derick Jiwan, Xianming Chen, Daniel Z. Skinner, Deven R. See

**Affiliations:** 1 Department of Plant Pathology, Washington State University, Pullman, Washington, United States of America; 2 United States Department of Agriculture -Agricultural Research Service, Crop Improvement and Protection Research Unit, Salinas, California, United States of America; 3 Department of Crop and Soil Sciences, Washington State University, Pullman, Washington, United States of America; 4 Stanford Center for Genomics & Personalized Medicine, Stanford University, School of Medicine, Palo Alto, California, United States of America; 5 United States Department of Agriculture-Agricultural Research Service, Wheat Health, Genetics, and Quality Research Unit, Pullman, Washington, United States of America; USDA, UNITED STATES

## Abstract

Stripe rust caused by *Puccinia striiformis* Westend. f. sp. *tritici* Erikks. is an economically important disease of wheat (*Triticum aestivum* L.). Hexaploid spring wheat landrace PI 480035 was highly resistant to stripe rust in the field in Washington during 2011 and 2012. The objective of this research was to identify quantitative trait loci (QTL) for stripe rust resistance in PI 480035. A spring wheat, “Avocet Susceptible” (AvS), was crossed with PI 480035 to develop a biparental population of 110 recombinant inbred lines (RIL). The population was evaluated in the field in 2013 and 2014 and seedling reactions were examined against three races (PSTv-14, PSTv-37, and PSTv-40) of the pathogen under controlled conditions. The population was genotyped with genotyping-by-sequencing and microsatellite markers across the whole wheat genome. A major QTL, *QYr*.*wrsggl1-1BS* was identified on chromosome 1B. The closest flanking markers were *Xgwm273*, *Xgwm11*, and *Xbarc187* 1.01 cM distal to *QYr*.*wrsggl1-1BS*, *Xcfd59* 0.59 cM proximal and *XA365* 3.19 cM proximal to *QYr*.*wrsggl1-1BS*. Another QTL, *QYr*.*wrsggl1-3B*, was identified on 3B, which was significant only for PSTv-40 and was not significant in the field, indicating it confers a race-specific resistance. Comparison with markers associated with previously reported *Yr* genes on 1B (*Yr64*, *Yr65*, and *YrH52*) indicated that *QYr*.*wrsggl1-1BS* is potentially a novel stripe rust resistance gene that can be incorporated into modern breeding materials, along with other all-stage and adult-plant resistance genes to develop cultivars that can provide durable resistance.

## Introduction

Stripe rust caused by *Puccinia striiformis* Westend. f. sp. *tritici* Erikss. is an economically important disease of wheat (*Triticum aestivum* L.). Especially in susceptible cultivars, stripe rust can cause yield losses as high as 100% when infection starts at the seedling stage and continues to develop throughout the growing season [1,2]. The cool and moist spring and early summer in the U.S. Pacific Northwest provide optimal conditions for stripe rust development early in the growing season. Additionally, the dry late summer and presence of wheat at different growth stages provide a perfect environment for urediniospores to carry over from late-harvested spring crops to survive and infect winter crops planted in the fall. Consequently, stripe rust is a reoccurring problem in the Pacific Northwest with frequent emergence of new races of the pathogen [2].

Genetic resistance is the most economical and environmentally friendly strategy to be applied against stripe rust. All-stage resistance and adult-plant resistance (APR) are the two major types of resistance described against wheat stripe rust [2]. All-stage resistance is detected in the seedling stage and continues to provide resistance throughout the plant’s growth stages. This kind of resistance is usually expressed at high levels, typically race-specific and qualitatively inherited, but frequently can be overcome by virulent races in the pathogen population due to strong selection pressure [3]. APR is expressed during later stages of plant development and can be race specific or race nonspecific. Race-nonspecific resistance is generally quantitatively inherited, often durable, and provides partial or slow-rusting resistance [2,4,5]. High-temperature adult-plant (HTAP) resistance is a type of APR that requires high temperature for plants to express resistance. The effectiveness of HTAP resistance increases as the plants grow old and as temperature increases [6]. Replacement of highly susceptible crop varieties with locally adapted resistant varieties possessing a combination of effective all-stage and adult-plant resistance will be the means of a sustainable solution to manage stripe rust [7,8]. More than 70 officially named stripe rust resistance genes and many unofficially named genes or quantitative trait loci (QTLs) have been identified and mapped to specific wheat chromosomal locations [9]. Effective resistance genes can be deployed in breeding programs to produce wheat cultivars with APR and/or combination of all-stage resistance to wheat rust [10]. Exploitation of known resistance in local and global breeding materials with the help of marker assisted selection has facilitated efficient breeding for resistance [2,8].

In search of novel rust resistance, a total of 652 spring wheat landrace accessions from 54 countries previously screened for resistance to the stem rust pathogen (race Ug99) [11] were tested for resistance to current races of the stripe rust pathogen in Pullman and Mt. Vernon, WA in 2011 and 2012. Thirty of the tested landraces had dual resistance to stem rust and stripe rust [12]. New and diverse resistant germplasm of both global and regional importance may be developed from landraces with resistance to stem rust and stripe rust. Genetic analysis of the landraces based on SSR (simple sequence repeat) and SNP (single nucleotide polymorphism) markers identified genetically diverse germplasm with resistance to rusts [12]. Germplasm from diverse genetic backgrounds potentially have different genes contributing to rust resistance. One of the landraces that was highly resistant to stripe rust in Washington during 2011 and 2012 was PI 480035, which was originally from Ethiopia. The objective of this research was to identify quantitative trait loci (QTL) for stripe rust resistance in PI 480035.

## Materials and methods

### Plant materials

PI 480035, which was resistant to stripe rust in the field in 2011 and 2012 (Sthapit et al. 2014), was used as the male parent to cross with the susceptible Australian spring wheat line, “Avocet Susceptible” (AvS). Accession PI 480035 is a hexaploid spring wheat landrace from the National Small Grains Collection (NSGC) that was originally collected from Shewa, Ethiopia in 1983. AvS has been used as a recurrent parent for developing near-isogenic lines for more than 20 stripe rust resistance genes [13]. The line is susceptible to most isolates of *P*. *striiformis* f. sp. *tritci* in the U.S. and other countries [14, 15]. The F_3:4_ families were developed in the greenhouse by single seed descent from F_2_ plants. F_3:4_ families were planted and evaluated for stripe rust resistance to natural inoculum in 2013 at Whitlow Farm, Pullman, WA. From the F_3:5_ seeds harvested from the field, single seeds were planted in the greenhouse to develop F_5:6_ recombinant inbred lines (RIL). F_5:6_ lines were used for further phenotyping and genotyping.

### Field experiments

RIL and parental lines were evaluated for stripe rust resistance in 2013 and 2014. In 2013, F_3:4_ families were planted in Pullman, WA on May 2^nd^. In 2014, F_5:6_ RIL were planted in Pullman, WA and Mt. Vernon, WA on April 18^th^ and May 1^st^, respectively. Seeds were planted in rows of 0.5 meter spaced at 30 cm. In 2014, three replications were planted in randomized complete block design in both locations. AvS was planted as spreader rows around the plots and was planted every 20 rows to enhance uniform rust development throughout the field.

Infection type (IT) and disease severity (DS) under natural infection of *P*. *striiformis* f. sp. *tritici* in the fields were scored two to three times (tillering, Zadoks stage 21–25; booting to heading, Zadoks 40–60; and heading to flowering stages, Zadoks 59–70) during the growing seasons. In 2014, spreader rows in Pullman, WA were artificially inoculated to build up enough inoculum in the screening nursery during the growing season, as dry weather did not allow formation of enough natural inoculum. Infection types based on the 0 to 9 scale [16] and DS as percentage of plant foliage infected in the row were visually scored. The area under disease progress curve (AUDPC) was calculated for the parents and each line of the population using the formula AUDPC = ∑_*i*_[(*x*_*i*_ + *x*_*i+1*_)/2]*t*_*i*_, where *x*_*i*_ is the DS on date *i*, *t*_*i*_ is the time in days between dates *i* and *i* +1. Relative area under disease progress curve (rAUDPC) based on DS was calculated for each plot relative to the mean AUDPC values of susceptible check AvS. For 2014, ITs, DS, and rAUDPC were averaged from three replicates for each line. ITs, DS, and rAUDPC from 2013 and mean ITs, DS, and rAUDPC from 2014 were used for QTL analysis.

### Greenhouse experiments

RIL and parental lines were also evaluated at the seedling stage for stripe rust resistance using three races of the pathogen; PSTv-14 (virulent to *Yr1*, *Yr6*, *Yr7*, *Yr8*, *Yr9*, *Yr17*, *Yr27*, *Yr43*, *Yr44*, *YrTr1*, *YrExp2*, *YrTye*), PSTv-37 (virulent to *Yr6*, *Yr7*, *Yr8*, *Yr9*, *Yr17*, *Yr27*, *Yr43*, *Yr44*, *YrTr1*, *YrExp2*), and PSTv-40 (virulent to *Yr6*, *Yr7*, *Yr8*, *Yr9*, *Yr10*, *Yr24*, *Yr27*, *Yr32*, *Yr43*, *Yr44*, *YrTr1*, *YrExp2*). PSTv-14 and PSTv-40 were predominant races in the Pacific Northwest, and PSTv-37 was the most widely distributed race of *P*. *striiformis* f. sp. *tritici* in the US in recent years [14,15]. Fresh urediniospores were produced on the leaves of the susceptible spring wheat line AvS as described by Chen and Line [17,18]. A set of 18 *Yr* single-gene line differentials [14] were also inoculated in order to confirm the race of *P*. *striiformis* f. sp. *tritici*. Fresh urediniospores were collected three to five times from about 16 days after inoculation until adequate harvest. Collected urediniospores were dried in a desiccator at 4°C and used for inoculation within a month.

F_5:6_ seeds of RIL and parents were planted in 72-well trays with five seeds per line and grown in a greenhouse. At 2-leaf stage, seedlings were inoculated with urediniospores of PSTv-14, PSTv-37, or PSTv-40 separately as described previously. Inoculated seedlings were incubated in a dark dew chamber at 10°C for approximately 24 h and transferred to a growth chamber with a diurnal temperature cycle gradually changing from between 4°C at 2:00 am and 20°C at 2:00 pm and 8 h dark/16 h light as described by Chen and Line [17,18]. Infection types were recorded on inoculated seedlings at 18 to 21 days after inoculation based on the 0 to 9 IT scale for stripe rust [16]. Recorded ITs were used for QTL analysis.

### Genotyping by sequencing

Seeds of the parental lines and F_6_ RIL were planted in 96-well trays with one seed per line. At two to three-leaf stage, leaf tissue was collected and lyophilized for about three days. Total genomic DNA was extracted and purified in a BioSprint 96 workstation with a BioSprint 96 DNA Plant kit (QIAGEN, Valencia, CA, USA). DNA was quantified using Quant-IT^TM^ PicoGreen® dsDNA Assay Kit (Thermo Fisher Scientific, Waltham, MA, USA) in Synergy 2 Multi-Mode Reader (BioTek, Winooski, VT, USA) and was normalized to 20 ng/μL using QIAgility (QIAGEN, Valencia, CA, USA). Extracted DNA was used for genotyping-by-sequencing (GBS) and run SSR markers.

GBS libraries were constructed using a combination of restriction enzymes *Pst*I and *Msp*I. The double restriction digestions, adapter ligation, and PCR multiplexing and amplification were performed according to the protocol provided by Mascher et al. [19]. Amplified PCR (polymerase chain reaction) products were pooled and cleaned up using the QIAquick PCR Purification Kit (QIAGEN, Valencia, CA, USA). Size selection of fragments at approximately 200 bp was done on E-Gel® iBase^TM^ and E-Gel® Safe Imager^TM^ Combo Kit using E-Gel® SizeSelect^TM^ Agarose Gel (2%) (Thermo Fisher Scientific, Waltham, MA, USA). Fragments from size selection were retrieved in ddH_2_O and analyzed in Agilent 2100 Bioanalyzer using Agilent High Sensitivity DNA Kit (Agilent Technologies, Santa Clara, CA, USA) for size distribution and quantification. Required amount of size selected library was used for further steps in sequencing. Ion sphere particle emulsion PCR was conducted on Ion OneTouch^TM^ 2 System (Thermo Fisher Scientific, Waltham, MA, USA) and template ion sphere particles were enriched on Ion OneTouch^TM^ ES following the protocol from Ion PI^TM^ Template OT2 200 Kit V2 User Guide (Thermo Fisher Scientific, Waltham, MA, USA). Percentage of templated ion sphere particles (ISPs) was determined by Ion Sphere^TM^ Quality Control Assay on Qubit® 2.0 Fluorometer (Thermo Fisher Scientific, Waltham, MA, USA). Libraries with optimum percent of template ion spheres were used for sequencing on Ion Proton^TM^ Sequencer following the protocol from Ion PI^TM^ Sequencing 200 Ki t V3 User Guide (Thermo Fisher Scientific, Waltham, MA, USA). Samples were loaded on Ion PI^TM^ Chip v2 for sequencing. The whole population was sequenced in one Ion Proton run. Each parent genotype was replicated in four wells for sequencing in order to maximize the number of GBS tags for the population. Sequences from all the samples of each parent were merged before data analysis.

### GBS data analysis

The Ion Proton^TM^ raw sequence data obtained via GBS had individual sample sequences assigned to their respective barcodes. The GBS sequences were trimmed to 75 bp and only sequences that started with the *Pst*I site were retained for further analysis. The above preprocessing followed by identification of SNPs and sequence homology comparison with the draft genome assembly of bread wheat [20] was conducted using the “GIO” analysis pipeline written in-house (unpublished). The genotype calls for co-dominant markers and dominant markers for both parents in the population were generated. For the whole population, GBS markers were called only if present in 20% to 80% of the population. There were 2,490 co-dominant markers and 22,142 (10,052 present in PI 480035 and 12,090 present in AvS) dominant markers. Twenty four genotypes having more than 60% of missing data were excluded from the analysis. In the remaining 88 individuals, in total, there was 33.37% of missing data for co-dominant markers. Imputation of missing data for co-dominant markers was performed using a custom script that uses missForest package in R [21]. Imputed GBS data was combined with SSR data for further analysis. Only codominant GBS markers were used for linkage and QTL analysis.

### SSR marker analysis

For the AvS × PI 480035 population, wheat SSR markers (microsatellites) with at least two from each arm of the 21 wheat chromosomes were selected for genotyping. Microsatellite regions were amplified by PCR with M13-tailed (CACGACGTTGTAAAACGAC) forward primers and M13 dye labeled primers. PCR was performed in 12-μL reactions containing 75 to 100 ng of genomic DNA, 1.2 μL 10x standard *Taq* polymerase buffer (BioLabs, MA, USA), 1 unit of *Taq* DNA polymerase, 12 mM MgCl2 (BioLabs), 2.4 μM dNTPs, 3 μM of reverse primer, 0.6 μM of forward primer with M13 tail at the 5’ end, and 2.4 μM of M13 primer labeled with fluorochrome (Applied Biosystems, Foster City, CA, USA). Thermocycler conditions included the following steps: initial denaturation at 94°C for 5 min, 42 cycles of denaturation at 94°C for 30 s, annealing at 50–61°C (specific to primers) for 45 s, extension at 72°C for 1 min. Final extension step was at 72°C for 10 min. Amplified PCR products were size separated by capillary electrophoresis on Applied Biosystems (Foster City, CA, USA) fragment analyzers (3130*xl* Genetic Analyzer or 3730*xl* DNA Analyzer). Raw data files from fragment analyzers were imported into GeneMarker v1.5 (SoftGenetics, State College, PA, USA) to analyze amplicon sizes. Polymorphic DNA fragments were scored for fragment lengths by GeneMarker v1.5.

### Linkage mapping and QTL analysis

Eighty-seven SSR markers and 2,490 GBS tags were combined for developing a linkage map (S1 Table and S2 Table). The GBS tags used in this project are publicly available, in FASTA format, from the Figshare repository (doi: 10.6084/m9.figshare.4831780). Scores for the markers were coded as ‘0’ or ‘2’ corresponding to the scores of the parents, PI 480035 and AvS, respectively, and heterozygotes were coded as ‘1’. Genetic linkage maps were constructed using the computer program JMP Genomics, Version 10 (SAS Institute Inc. Cary, NC, 1989–2007). The Kosambi mapping function [22] was used to estimate the centimorgan (cM) values. Each linkage group was assigned to a wheat chromosome or a chromosome arm based on matches of GBS tags with sequences from a draft genome sequence of bread wheat [20] and previously mapped SSR markers [23]. Linkage groups with less than three markers were not considered.

QTL analyses were performed on QTL Cartographer Version 2.5 [24] using composite interval mapping (CIM). QTL analysis was performed on IT scored for each race used (PSTv-14, PSTv-37, or PSTv-40), IT and DS scored in seedling and adult stages in the field, rAUDPC calculated for each environment in two field years, and also mean values for IT, DS, and rAUDPC for all seasons. A permutation of 1,000 (*P ≤ 0*.*05*) was used to determine the significance threshold for the detection of QTL.

## Results

### Stripe rust evaluation

**Fig 1:**
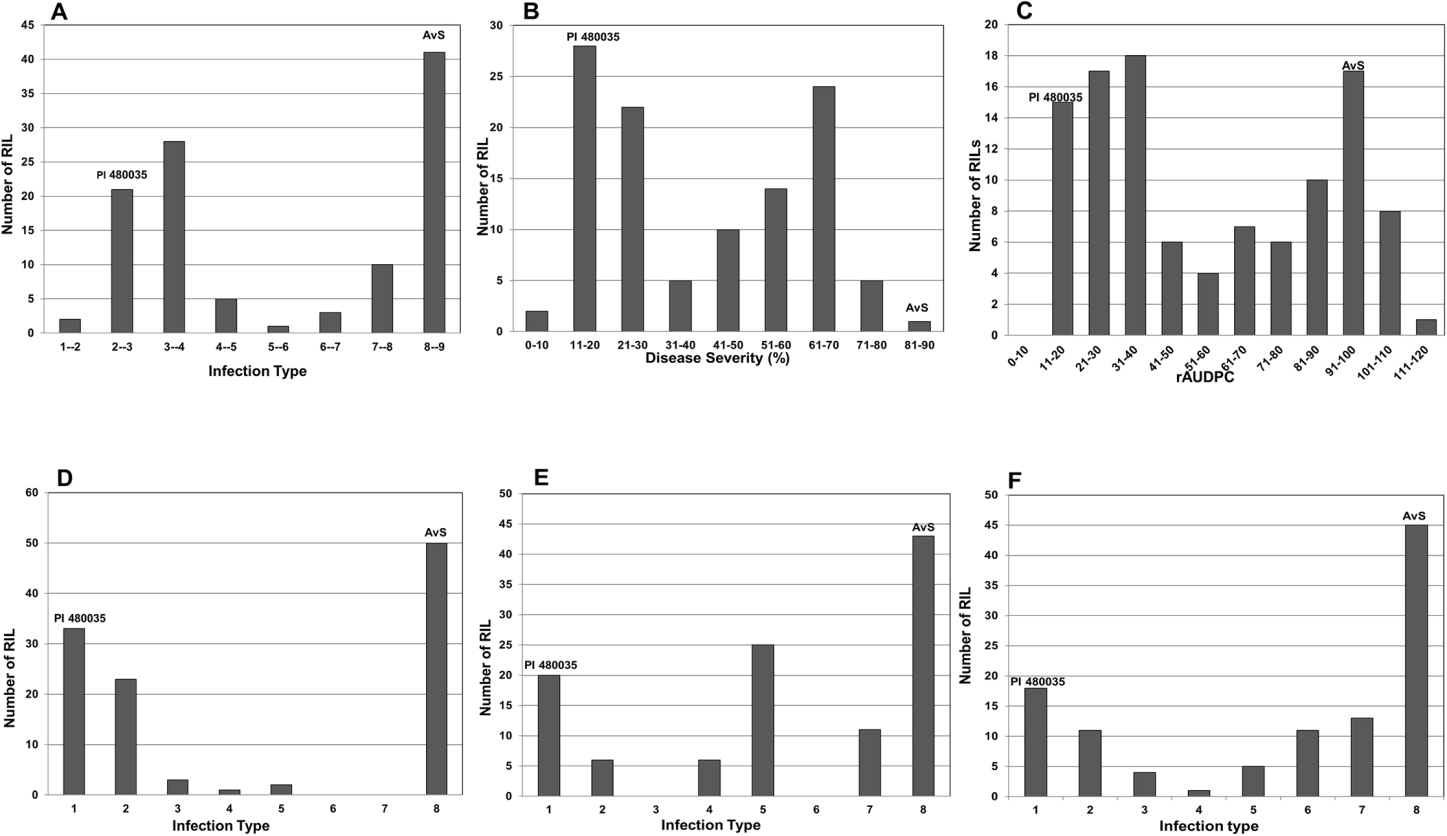
Histograms for the AvS × PI 480035 population for the distribution of seedling infection types (IT) for three *Puccinia striiformis* f. sp. *tritici* races and mean values of IT, disease severity (DS), and relative area under disease progress curve (rAUDPC) across all seasons and environments (Pullman 2013, Pullman 2014, and Mt. Vernon 2014). (A) Mean IT; (B) Mean DS; (C) Mean rAUDPC; (D) IT for race PSTv-14; (E) IT for race PSTv-37; and (F) IT for race PSTv-40.

In the field, ITs of the susceptible parent AvS were 8 to 9 (S3 Table) in each environment at all growth stages. The resistant parent PI 480035 scored IT 2 to 3 in the fields at all growth stages evaluated, with DS from 3 to 20%, and rAUDPC ranging from 9.6 to 25.6%. ITs for the entire AvS × PI 480035 population of 110 RILs ranged from 1 to 9, DS from 2 to 100%, and rAUDPC ranged from 6.4 to 135.4% in the field. The IT, DS scores, and rAUDPC from all environments were averaged for each line of the population into mean IT, mean DS, and mean rAUDPC values. The correlation between mean IT and mean DS was high with the Pearson Correlation (R) of 0.95. Distribution of mean IT, mean DS, and mean rAUDPC of the population in the environments were all bimodal (Fig 1A, 1B and 1C). The most predominant race of *P*. *striiformis* f. sp. *tritici* in Pullman in 2013 was PSTv-52 followed by PSTv-37. In 2014, the most predominant race in Pullman fields was PSTv-79 followed by PSTv-52, and in Mt. Vernon was PSTv-52 followed PSTv-37 (A.M.Wan and X.M. Chen, unpublished data).

In the greenhouse seedling experiments with PSTv-14, PSTv-37, and PSTv-40, ITs of 8 to 9 were observed for the susceptible parent AvS. The resistant parent, PI 480035, was scored IT of 1 for all three races. ITs for the whole population ranged from 1 to 8. Distribution of ITs of the population in the tests of PSTv-14 and PSTv-40 were bimodal indicating that a single locus was responsible for resistance (Fig 1D and 1F). Distribution of ITs for PSTv-37 was skewed towards higher IT indicating more susceptibility (Fig 1E).

### Genetic linkage mapping

Linkage analysis of segregating genetic markers mapped a total of 1366 GBS tags and 57 SSR markers into 35 linkage groups. The linkage groups were assigned to wheat chromosomes and chromosome arms based on previously published information on SSR markers and comparison of GBS markers against the draft genome reference sequence of bread wheat. The shortest linkage group was 7BS (22.3 cM) and the longest linkage group was 1B (406.1cM). The length of the whole genome was 4,052.8 cM. The average length of a linkage group was 115.8 cM and the average distance between two markers was 2.84 cM. Linkage groups from all wheat chromosomes were represented by one or more linkage groups except for chromosome 3D. The 35 linkage groups were used for the QTL analysis.

### QTL analysis

**Fig 2:**
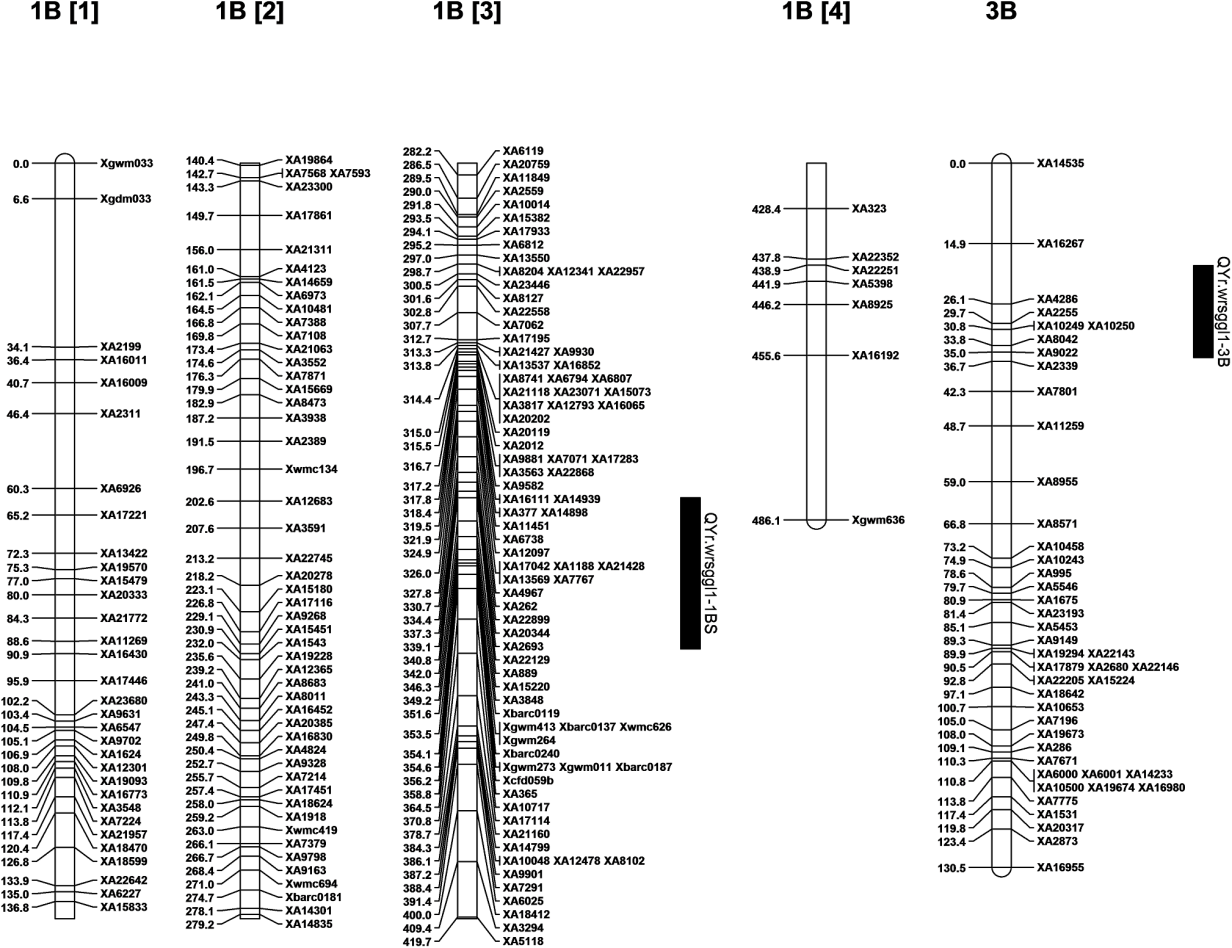
Chromosomal location of quantitative trait loci (QTLs) for stripe rust resistance in the AvS × PI 480035 population.

**Fig 3:**
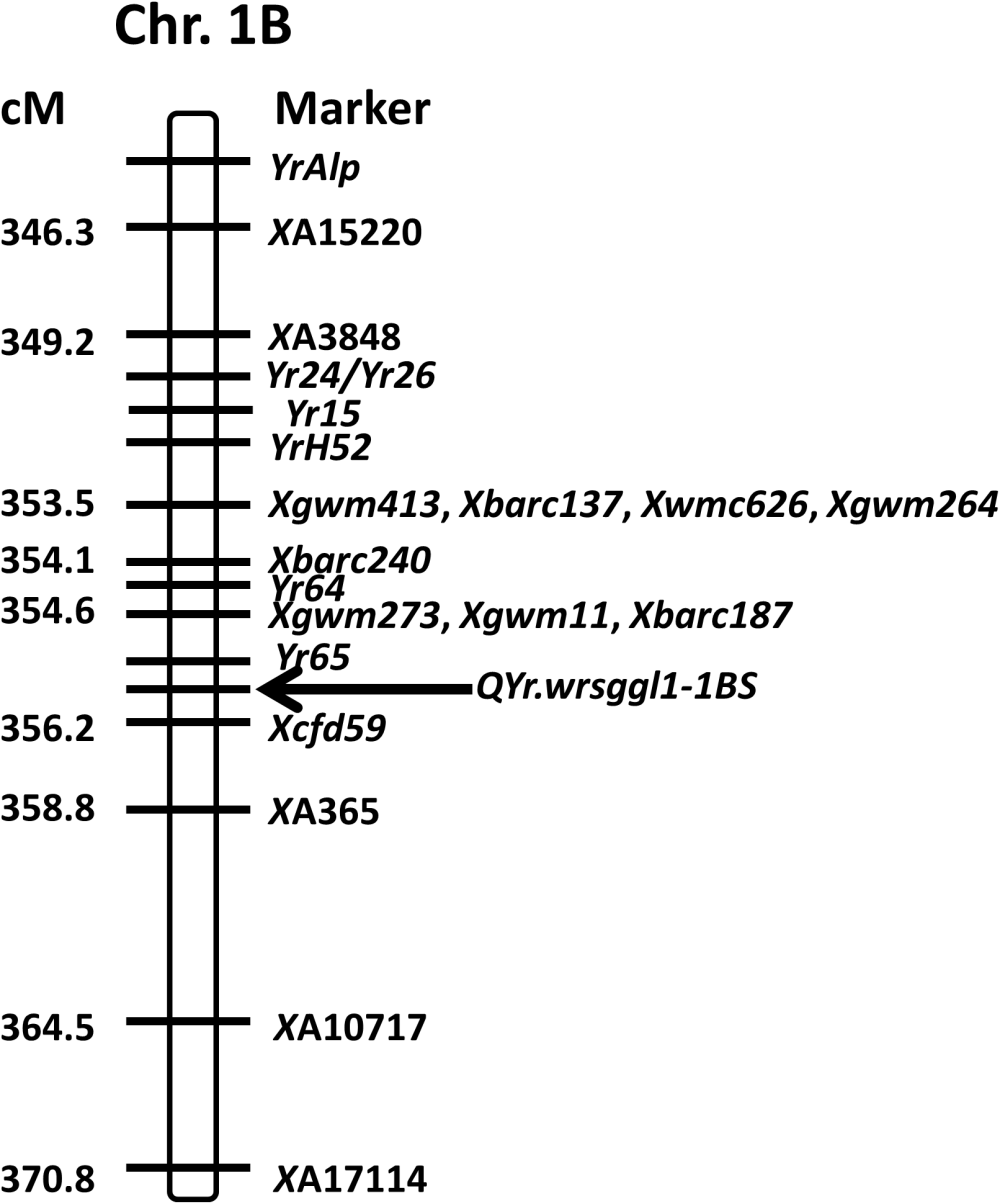
Chromosomal location of major quantitative trait locus (*QYr*.*wrsggl1-1BS*) for stripe rust resistance in the AvS × PI 480035 population in comparison to other stripe rust resistance genes previously known to be located on chromosome 1BS. Positions of the resistance genes are based on the linked markers.

**Fig 4:**
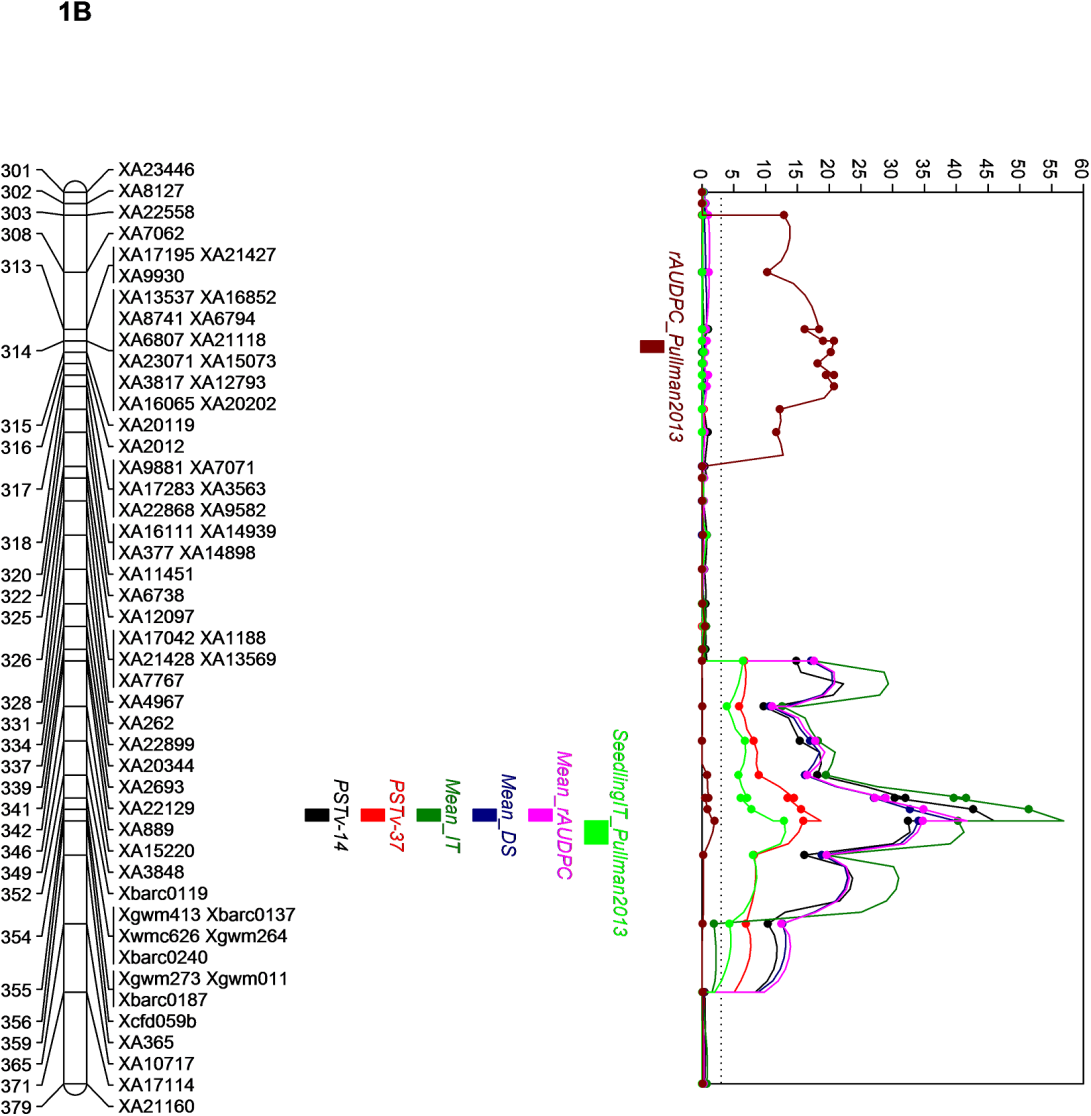
Quantitative trait locus, *QYr*.*wrsggl1-1BS* for stripe rust resistance in chromosome 1B.

**Fig 5:**
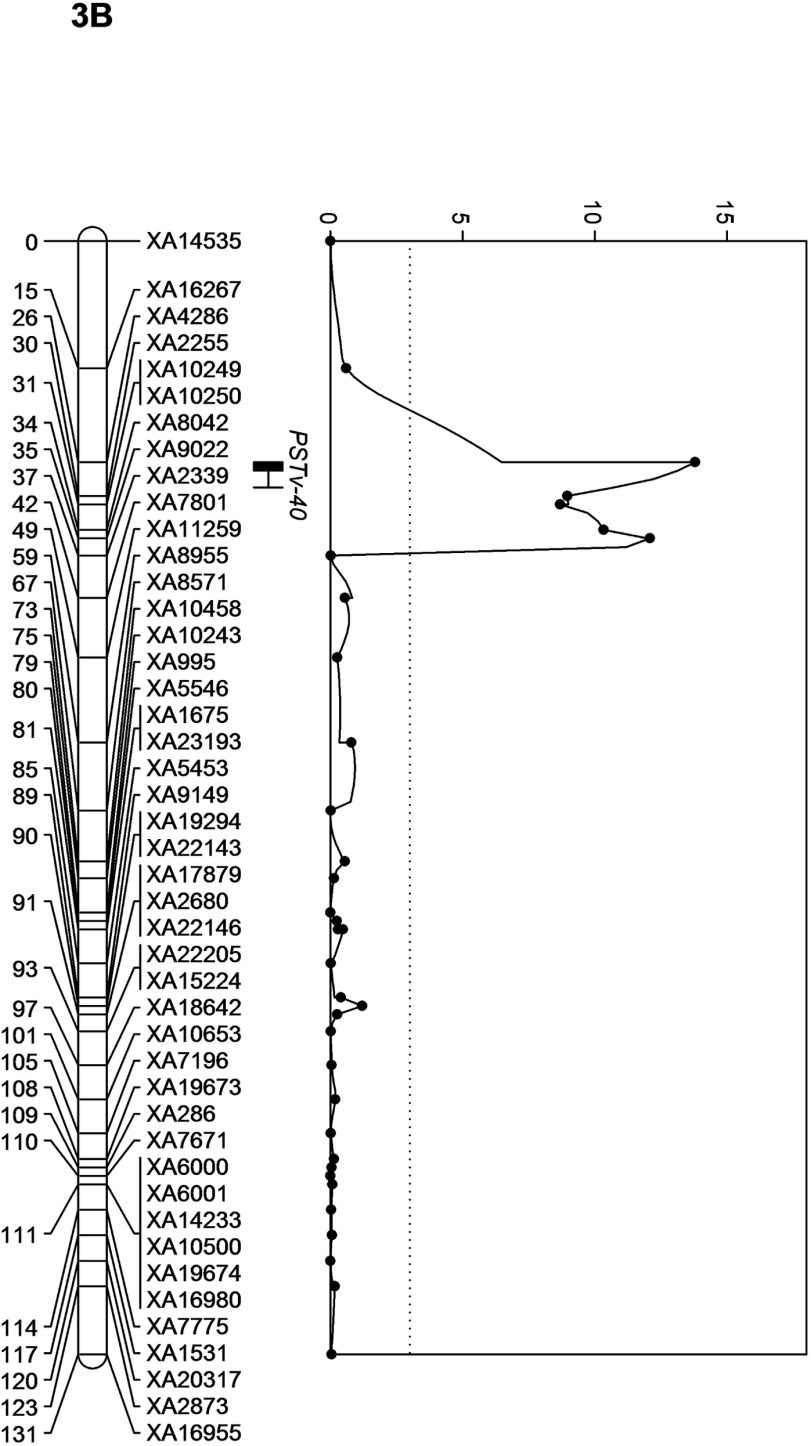
Quantitative trait locus, *QYr*.*wrsggl1-3B* for stripe rust resistance in chromosome 3B.

**Fig 6:**
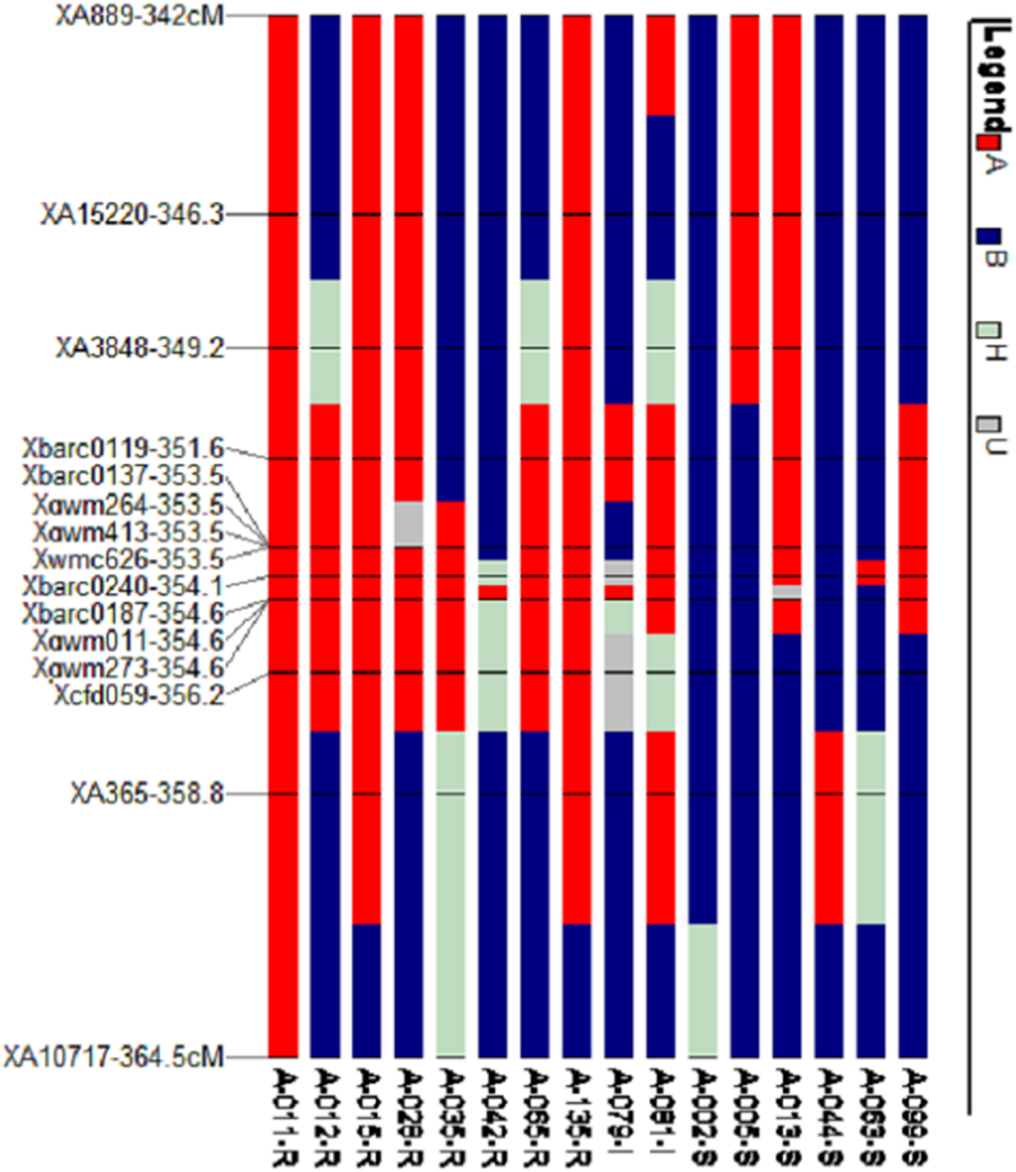
Graphical genotypes of 16 F_7_ individuals from the AvS x PI 480035 population having different recombination pattern in the 1B QTL region. R, I, and S at the end of the individual names designate resistant (IT 0–3), intermediate (IT 4–6), and susceptible (IT 7–9) reaction of the plants to stripe rust race PSTv-14. A (red bar) refers to genotype from resistant parent (PI 480035), B (blue bar) refers to genotype from susceptible parent (AvS), H is heterozygote, U is missing data.

The QTL analysis identified a major QTL for stripe rust resistance on chromosome 1B and another QTL significant for race PSTv-40 was identified on chromosome 3B (Table 1). The QTL on 1B was designated as *QYr*.*wrsggl-1B* and was significantly associated with IT, DS, and rAUDPC values in all field tests as well as with IT to races PSTv-14 and PSTv-37 in the seedling tests. It was not significant for race PSTv-40. The highest LOD score was 65.2 for the IT scored at adult stage in Pullman in year 2014 and explained 91% (R^2^ of 0.91) of the phenotypic variation. The LOD score for races PSTv-14 and PSTv-37 were 46.0 and 18.7, respectively, explaining 84% and 48% of the phenotypic variation in the population. The QTL on 1B was significant for all means from all locations with LOD scores of 56.9 for IT, 39.9 for DS, and 41.7 for rAUDPC accounting for 88, 74, and 77% of phenotypic variation, respectively. The highest peak position of the 1B QTL was at 355.61 cM for all the tested traits except for seedling IT at Pullman in 2013 (357.21 cM) and rAUDPC at Pullman in 2013 (314.41 cM). The QTL region was saturated with SSR markers and was flanked by *Xgwm273*, *Xgwm11*, and *Xbarc187* at a distance of 1.01 cM distally, and by *Xcfd59* at 0.59 cM, and *XA365* at 3.19 cM proximally (Figs 2, 3, 4 and 5). Graphical genotyping of the markers linked to the 1B QTL on 16 individuals showing different recombination pattern in the region demonstrated that the QTL is located between *Xcfd59* and *Xgwm273*, *Xgwm11*, and *Xbarc187* (Fig 6).

**Table 1:** Quantitative trait loci associated with stripe rust resistance in the AvS × PI 480035 population.

Trait	Environment	Chromosome	LOD^a^	Position (cM)^b^	R^2(^^c^^)^
Seedling IT^d^	PSTv-14	1B	46.0	355.61	0.84
Seedling IT	PSTv-37	1B	18.7	355.61	0.48
Seedling IT	PSTv-40	3B	13.8	26.11	0.43
Seedling IT	Pullman 2013	1B	13.1	357.21	0.24
Seedling IT	Pullman 2014	1B	56.9	355.61	0.89
Seedling IT	Mt. Vernon 2014	1B	56.5	355.61	0.32
Adult IT^e^	Pullman 2013	1B	20.2	356.21	0.59
Adult IT	Pullman 2014	1B	65.2	355.61	0.91
Adult IT	Mt. Vernon 2014	1B	34.4	355.61	0.39
Adult DS^f^	Pullman 2013	1B	19.3	355.61	0.45
Adult DS	Pullman 2014	1B	42.6	355.61	0.83
Adult DS	Mt. Vernon 2014	1B	24.7	355.61	0.68
rAUDPC^g^	Pullman 2013	1B	20.8	314.41	0.53
rAUDPC	Pullman 2014	1B	46.1	355.61	0.86
rAUDPC	Mt. Vernon 2014	1B	33.4	355.61	0.73
Mean IT^h^	all seasons	1B	56.9	355.61	0.88
Mean DS^h^	all seasons	1B	39.9	355.61	0.74
Mean rAUDPC^h^	all seasons	1B	41.7	355.61	0.77

^a^Logarithm of odds.

^b^Chromosomal location of QTL in cM.

^c^Percentage of phenotypic variation explained by QTL.

^d^Infection type scored at seedling stage.

^e^Infection type scored at adult plant stage.

^f^Disease severity scored at adult plant stage.

^g^Relative area under disease progress curve relative to the susceptible check AvS.

^h^Mean values for scores from all years and environments.

Another QTL was identified on chromosome 3B, which was significant only for seedling IT for race PSTv-40. The QTL was designated as *QYr*.*wrsggl-3B*. The LOD score for this QTL was 13.8 and it explained 43% of the phenotypic variation. The QTL lies on the GBS marker *XA4286* and was flanked by *XA2255* at 3.59 cM proximally and at *XA16267* by 11.21 cM distally (Fig 2).

## Discussion

Resistant spring wheat landrace PI 480035, originally collected from Ethiopia and deposited in the USDA Small Grains Collection in 1983 (http://www.ars-grin.gov) was crossed with susceptible spring wheat AvS to develop a biparental population. QTLs associated with stripe rust resistance in the field environment and at seedling stage to races PSTv-14, PSTv-37, and PSTv-40 were identified. A major QTL, *QYr*.*wrsggl-1B*, located on chromosome 1B was significantly associated with IT, DS, and rAUDPC for stripe rust resistance in all environments, as well as for races PSTv-14 and PSTv-37 under controlled conditions. When mean values from all environments were compared between the RILs with and without the QTL, it reduced IT scores from 8 to 3, DS values from 61.09 to 23.8%, and rAUDPC from 85.46 to 32.8%. A QTL on chromosome 3B, *QYr*.*wrsggl-3B*, was associated with resistance to race PSTv-40 and reduced seedling IT scores from 8 to 3. The RILs carrying both QTLs were highly resistant at all environments and to all three races tested.

Comparing positions of the flanking SSR markers from the 1B linkage map to the consensus microsatellite map [23], the QTL is located in 1BS near the centromere. Many important stripe rust resistance genes have been previously reported to be located on wheat chromosome 1B including *Yr10* [25], *Yr15* [26], *Yr21* [27,28], *Yr24/26/CH42* [29], *Yr29* [30] on 1BL, and *Yr64*, *Yr65* [31] on 1BS. *Yr9* [32] is located on 1RS of the 1BL.1RS wheat-rye translocation chromosome. Some temporarily designated *Yr* genes mapped to 1B include *YrAlp* [33], *YrC142* [34], *YrCN17* [35] derived from *Secale cereale*, *YrExp1* [36], *YrH52* [26], and *YrR212* [35]. There are some QTL for stripe rust resistance that have been mapped to 1B including *QYr*.*sun-1B* [37], *QPST*.*jic-1BL* [38], *QYrco*.*wpg-1B*.*1* and *QYrco*.*wpg1B*.*2* [39]. All these reports indicate that chromosome 1B is rich of genes for stripe rust resistance.

Wang et al. [25] mapped *Yr10* at 25.9 cM distal to *Xgwm11*. In the AvS × PI 480035 population, X*gwm11* was only 1.01 cM proximal to the QTL, indicating that it is not *Yr10*. Murphy et al. [40] showed that *Xbarc8* and *Xgwm413* are completely linked to *Yr15*. In the AvS × PI 480035 population, neither *Xbarc8* nor *Xgwm413* showed the diagnostic allele indicative of the presence of *Yr15*. Furthermore, the 1BS gene is not effective against race PSTv-40, whereas *Yr15* is effective against all races identified in the US including PSTv-40 [14,15]. Wang et al. [29] mentioned that *Yr26* was 3.2 cM proximal to *Xgwm11*. *Yr24* and *Yr26* are likely the same gene for stripe rust resistance [41]. In the AvS × PI 480035 population, *Xgwm11* was 1.01 cM distal to the QTL. According to Cheng et al. [31], *Yr64* was 3.5 cM proximal to *Xgwm413* and distal to *Xcfd59* by 6.9 cM as well as distal to *Xgwm273* by 7.3 cM. *Yr65* was 2.1 cM distal to *Xgwm11* and 4.9 cM proximal to *Xgwm273* [31]. According to Peng et al. [26], *YrH52* was 1.3 cM distal to *Xgwm413* and 2.7 cM proximal to *Xgwm273*. Lin and Chen [33] indicated that *YrAlp* was 16.3 cM distal to *Xgwm11*. In the AvS × PI 480035 population, the QTL on chromosome 1B was 2.11 cM proximal to *Xgwm413*, 1.01 cM proximal to *Xgwm273* and *Xgwm11*, and 0.59 cM distal to *Xcfd59*. Based on the positions of *Xcfd59*, *Xgwm273*, *Xgwm413*, and *Xgwm11* in the AvS × PI 480035 population and comparing with maps for other *Yr* genes in this region, the identified QTL (*QYr*.*wrsggl-1BS*) was located proximal to *Yr65* (Fig 3). *Yr65* was reported from durum (tetraploid) wheat accession PI 480016 originally collected from Ethiopia [31]. Another *Yr* gene close to the QTL *QYr*.*wrsggl-1BS* was *Yr64* which also was from tetraploid wheat. *Yr64* was reported from durum wheat accession PI 331260 originally collected from Ethiopia [31]. Alleles of SSR markers flanking *Yr64* and *Yr65* were compared to the alleles of PI 480035 (S4 Table). For *Yr64*, the PI 480035 allele for flanking marker *Xgwm413* was the same as in PI 331260 (123 bp) but *Xgwm498* was monomorphic with an allele of 175 bp for both AvS and PI 480035 indicating the QTL region was genetically different. For *Yr65*, the allele was different for both AvS (221 bp) and PI 480035 (211 bp) from AvS (208 bp) and PI 480016 (206 bp) as mentioned by Cheng et al. [31], indicating different region amplification or different alleles. The flanking marker, *Xgwm18*, for *Yr65* was monomorphic with the allele of 207 bp for both AvS and PI 480035 indicating that the QTL was different from *Yr65*.

The QTL, *QYr*.*wrsggl-3B*, located on chromosome 3B, was significant only for resistance against race PSTv-40. It was not significant in the field environments indicating that it was a race-specific resistance and was not able to protect against other races present in the fields. Comparing the virulence formulae of the three *P*. *striiformis* f. sp. *tritici* races tested in this study, PSTv-40 is not virulent to *Yr17* for which PSTv-14 and PSTv-37 were virulent. The QTL in the AvS × PI 480035 population was not *Yr17*, as *Yr17* is located in chromosome 2AS and it was transferred to wheat from *Aegilops ventricosa* introgression [42]. On chromosome 3B, already reported resistance genes and loci are *Yr30* [43], *Yr4* [44], *Yr57* [45], *YrS* [46] and *Yrns-B1* [47]. *Yr30* confers adult plant resistance and is closely linked to *Sr2* and *Lr27*, and *Sr2* is associated with pseudo-black chaff. *QYr*.*wrsggl-3B* was significant only for resistance to PSTv-40 in the seedling test and was not associated to pseudo-black chaff, indicating that it was not *Yr30*. As the 3B QTL region was not saturated with other molecular markers as SSRs and SNPs, the QTL could not be compared to other previously reported stripe rust resistance genes on 3B. The QTL was exactly located over the GBS marker *XA4286*. When this marker and the flanking markers, *XA2255* and *XA16267*, were compared with the draft genome sequence of bread wheat [20], they matched with the non-genic regions, and when NCBI BLAST performed, no informative results were obtained except for genomic scaffold or contigs. The limited contribution of this QTL towards stripe rust and its ineffectiveness in the field minimizes its importance in the US Pacific Northwest.

The major QTL identified, *QYr*.*wrsggl-1B*, was effective at all environments, at all stages indicating that it confers all-stage resistance. However, it was not effective to race PSTv-40, indicating that it confers race-specific resistance. Similarly, *QYr*.*wrsggl-3B* was not significant in field environments and races tested other than PSTv-40 demonstrating its race specificity as well. These QTL can be integrated with other adult-plant and all-stage resistance genes to deploy them effectively.

## Supporting information

S1 Table List of GBS (genotyping-by-sequencing) markers used for linkage and QTL mapping for AvS x PI 480035 population. The table includes sequence of the markers, position of the SNPs, and chromosome/s in which the marker sequence homology was identified by comparison with the draft genome assembly of bread wheat [19].
(XLSX)

S2 Table Genotypic data of the AvS x PI 480035 population used for linkage mapping. (XLSX)

S3 Table Phenotypic data of the AvS x PI 480035 population used for QTL mapping. (XLSX)

S4 Table Alleles for SSR (simple sequence repeat) markers flanking genes *Yr64* and *Yr65* (Cheng et al. 2014) on the parental lines of AvS x PI 480035 population. (DOCX)
