# Supplementary material for: Mapping genes for resistance to stripe rust in spring wheat landrace PI 480035
Source: PLoS One. 2017 May 19;12(5):e0177898. doi: 10.1371/journal.pone.0177898 (PMC5438115; doi:10.1371/journal.pone.0177898)
Supplement: S4 Table — (DOCX) [file pone.0177898.s004.docx]

**S4 Table. Alleles for SSR (simple sequence repeat) markers flanking genes *Yr64* and *Yr65* (Cheng et al. 2014) on the parental lines of AvS x PI 480035 population.**

|  | Marker alleles in bp^a^ | | | | |
| --- | --- | --- | --- | --- | --- |
|  | *Yr64* | |  | *Yr65* | |
| Accession | *Xgwm413* | *Xgwm498* |  | *Xgwm11* | *Xgwm18* |
| Avocet S (AvS) | 121 | 175 |  | 221 | 207 |
| PI 480035 | 123 | 175 |  | 211 | 207 |
|  |  |  |  |  |  |
| PI 331260 (*Yr64*)^b^ | 123 | 173 |  | 206 | 205 |
| PI 480016 (*Yr65*)^b^ | 121 | 173 |  | 206 | 205 |
| AvS^b^ | 121 | 175 |  | 208 | 207 |

^a^Allele size includes 19-bp M13 tail (CACGACGTTGTAAAACGAC) from forward primers

^b^Parental lines and their alleles as reported in Cheng et al. 2014
